# Modelling excess mortality among breast cancer patients in the North East Region of Peninsular Malaysia, 2007–2011: a population-based study

**DOI:** 10.1186/s12889-019-8113-2

**Published:** 2019-12-30

**Authors:** Tengku Muhammad Hanis, Najib Majdi Yaacob, Suhaily Mohd Hairon, Sarimah Abdullah, Noorfariza Nordin, Noor Hashimah Abdullah, Mohd Faiz Md Ariffin

**Affiliations:** 1 Unit of Biostatistics and Research Methodology, School of Medical Sciences, Health Campus, Universiti Sains Malaysia, 16150 Kubang Kerian, Kelantan Malaysia; 2 Department of Community Medicine, School of Medical Sciences, Health Campus, Universiti Sains Malaysia, 16150 Kubang Kerian, Kelantan Malaysia; 3 Kelantan State Health Department, Aras 5, Wisma Persekutuan Kota Bharu, Jalan Bayam, 15590 Kota Bharu, Kelantan Malaysia

**Keywords:** Breast cancer, Relative survival, Excess hazard, Excess mortality

## Abstract

**Background:**

Measurement of breast cancer burden and identification of its influencing factors help in the development of public health policy and strategy against the disease. This study aimed to examine the variability of the excess mortality of female breast cancer patients in the North East Region of Peninsular Malaysia.

**Methods:**

This retrospective cohort study was conducted using breast cancer data from the Kelantan Cancer Registry between 2007 and 2011, and Kelantan general population mortality data. The breast cancer cases were followed up for 5 years until 2016. Out of 598 cases, 549 cases met the study criteria and were included in the analysis. Modelling of excess mortality was conducted using Poisson regression.

**Results:**

Excess mortality of breast cancer varied according to age group (50 years old and below vs above 50 years old, Adj. EHR: 1.47; 95% CI: 1.31, 4.09; *P* = 0.004), ethnicity (Malay vs non-Malay, Adj. EHR: 2.31; 95% CI: 1.11, 1.96; *P* = 0.008), and stage (stage III and IV vs. stage I and II, Adj. EHR: 5.75; 95% CI: 4.24, 7.81; *P* < 0.001).

**Conclusions:**

Public health policy and strategy aim to improve cancer survival should focus more on patients presented at age below 50 years old, Malay ethnicity, and at a later stage.

## Background

Breast cancer is the leading cause of mortality and morbidity among women globally [1]. About 24% of total breast cancer cases worldwide were accounted for in the Asia Pacific region in 2012 [2]. A study in 2014 reported that breast cancer accounted for 25% of cancer-related deaths in Malaysia which was higher than neighbouring countries such as Indonesia (22%), Singapore (20%) and Brunei (17%), while in term of mortality to incidence rate ratio Malaysia was at 0.49 whereas other neighbouring countries such as Indonesia (0.41), Brunei (0.23), and Singapore (0.24) had a lower ratio [2]. Additionally, according to the Malaysian National Cancer Registry report, breast cancer was the most common cancer among Malaysian between 2007 and 2011, by which 99% of cases were female [3].

The survival statistics are the most commonly used measures to reflect the prognosis and the burden of cancer [4]. There are two approaches for the survival analysis; the relative survival approach and the cause-specific survival approach. The use of relative survival approach in a population-based cancer study has been considered as a standard practice [5]. The main challenges of conducting the population-based study are the availability and the quality of the data. The cause of death in most of the cancer registries is not reliable and sometimes is not available at all. Thus, a cause-specific survival approach is not appropriate in this condition, and the relative survival approach is justified since the information on the exact cause of death is not necessary for this approach.

Over the last 10 years, in Malaysia, most of the research conducted to study the burden of breast cancer, and its prognostic factors used the local hospital registry data, and the cause-specific survival approach [6–13] with only a few were population-based studies [11–13]. Given the scarcity of breast cancer studies among Malaysian residents at the population level, this study was conducted to measure the prognostic factors of excess mortality among female breast cancer patients in one of the regions in Malaysia using data from a population-based cancer registry.

## Methods

### Study site and population

North-East Region of Peninsular Malaysia consists of three states; Kelantan, Terengganu, and Pahang. This study was conducted in Kelantan state where the majority of the residents were Malay (94.6%), followed by Chinese (3.3%), Indian (0.3%), and others (1.8%) [3]. In this study, two sources of data were used for relative survival approach namely 1) the expected population which was derived from the general population mortality data, and 2) the observed population which was derived from the breast cancer data.

### General population mortality data

General population mortality data for Kelantan was obtained from the Department of Statistics, Malaysia (DOSM). To be able to conduct the relative survival analysis, the general population mortality data must be in the form of a complete life table that matched breast cancer data by age, sex, and mortality year. However, the only available data from DOSM was in the form of the abridged life table. Consequently, the data need to be expanded to a complete life table. The general population mortality data for Kelantan between 2007 and 2016 were downloaded from the DOSM website (https://newss.statistics.gov.my/newss-portalx/ep/epLogin.seam) in the Microsoft Excel spreadsheet. Variables available in the spreadsheet were age, probability of dying between age x and age x + n, number of deaths between age x and age x + n, central mortality rate between age x and age x + n, survivors at exact age x, number of person-years lived between age x and age x + n, survival ratio, total number of person-years lived after exact age x, and life expectancy.

### Breast cancer data

Breast cancer data was obtained from the Kelantan Cancer Registry in Non-Communicable Disease (NCD) unit, Kelantan State Health Department. Kelantan Cancer Registry data includes all cancer cases notified by all hospitals in Kelantan, which consist of nine public hospitals; Hospital Raja Perempuan Zainab II, Hospital Pasir Mas, Hospital Tumpat, Hospital Machang, Hospital Tanah Merah, Hospital Tengku Anis, Hospital Gua Musang, Hospital Kuala Krai, Hospital Jeli, five private hospitals: KPJ Perdana Specialist Hospital, Kota Bharu Medical centre, An-Nisa’ Medical Centre, Telipot medical Centre and USAINS Hospital, and one university hospital; Hospital Universiti Sains Malaysia. The relevant information such as age at diagnosis, sex, ethnicity, cancer morphology, cancer staging, surgery, chemotherapy, radiotherapy, date of diagnosis, status, and date of death was assessed. The extracted data only included breast cancer cases diagnosed between 1st January 2007 and 31st December 2011.

### Study design and patient selection

This was a retrospective cohort study of female breast cancer using Kelantan Cancer Registry. All breast cancer cases were diagnosed with International Classification of Diseases for Oncology (ICD-O) codes C50 series. The inclusion criteria were that the cases must be diagnosed between 1st January 2007 and 31st December 2011, and a Kelantan resident. Additionally, male patients and patients with incomplete data of any variables were excluded. All breast cancer cases had a follow-up record until 31st December 2016. Out of 598 cases, 46 cases with missing information on cancer staging and three cases of male breast cancer were excluded from the study. Thus, 549 of breast cancer cases met the study criteria and were included in the analysis.

### Statistical analysis and software

This study used MORTPAK for Windows version 4.3 [14] for expansion of the abridged life Tables. R version 3.6.0 [15] was used for data cleaning and manipulation, descriptive statistics, univariable Poisson regression, multivariable Poisson regression.

### Expanding abridged life table

The abridged life tables of Kelantan population mortality were expanded into the complete life tables using the UNABR application in the MORTPAK software [14]. The UNABR application used the Heligman-Pollard model for this expansion. The variant of the model used in the UNABR was:

1 $$ 1\mathrm{qx}={A}^{{\left(x+B\right)}^c}+{De}^{-E{\left(\mathit{\ln}\ x-\mathit{\ln}\ F\right)}^2}+\frac{GH^x}{1+{GH}^x\ } $$

where 1qx denotes the probability of dying at a yearly interval, and A, B, C, D, E, F, G, and H were the parameters to be estimated. Several studies had agreed that the Heligman-Pollard model fits the Malaysian population considerably well [16–18]. Two variables needed for the UNABR application were age and probability of dying between age x and age x + n from the abridged life tables of Kelantan population mortality data. A complete life table produced from this application included variables; age in the yearly interval, central mortality rate between age x and age x + 1, probability of dying between age x and age x + 1, survivors at exact age x and life expectancy.

### Descriptive statistics

The numerical variables were checked for normal distribution visually by histogram and quantile-quantile plot (Q-Q plot). An approximation of a bell-shaped curved histogram and a 45° line in Q-Q plot were considered as a normally distributed variable. The numerical variables were presented in mean and standard deviation (SD) for a normally distributed variable, and in median and interquartile range (IQR) for a non-normally distributed variable. The categorical variables were presented in frequency and percentage (%). The survival time was presented in range, minimum value, maximum value, median, and IQR.

### Poisson regression

This analysis was conducted using a relsurv package [19] in R software. The analysis of excess hazard was carried out using Poisson regression as proposed by Dickman et al. [5]:

2 $$ \mathit{\ln}\left({u}_{jk}-{d}_{jk}^{\ast}\right)=\mathit{\ln}\left({y}_{jk}\right)+ x\beta +{\gamma}_k $$

where;

*u*_*jk*_ = number of deaths for observation j in interval k,

*y*_*jk*_ = person-time at risk for observation j in interval k,

${d}_{jk}^{\ast }$ = number of deaths in the expected population comparable to observation j in interval k,

*xβ* = a vector of covariate x assumed to be in multiplicative function with coefficient β,

*γ*_*k*_ = coefficient in time interval k.

In univariable Poisson regression, the survival times were split into several time intervals. Thus, the time intervals were set according to the recommendation of the United Kingdom and Ireland Association of Cancer Registries (UKIACR) which were monthly up to 6 months, 3-monthly up to 2 years, 6-monthly during 2 to 5 years, and yearly up to 10 years [20]. However, for variable radiotherapy and chemotherapy, different time intervals were used since the univariable models for both variables did not converge. The time intervals used were monthly up to 6 months, 3-monthly up to 2 years, 6-monthly during 2 to 5 years, yearly up to 7 years, and 3-yearly up to 10 years. All variables with a *p*-value below 0.25 were included in the multivariable Poisson regression.

In modelling the multivariable Poisson regression, the analysis was conducted using the time intervals recommended by the UKIACR. Variable with the highest p-value above 0.05 was removed one at a time. Once the variables for the Multivariable Poisson regression were confirmed, the time intervals were reduced to achieve a more parsimonious model. Models comparison were done using Deviance (−2Log-likelihood), Akaike Information Criterion (AIC), and the most significant *p*-value for each variable.

Finally, the final model was tested for all possible two-way interactions between the variables, non-proportional excess hazard, and overdispersion. A p-value below 0.05 indicates a significant two-way interaction term. For the non-proportional excess hazard model, the interaction term between variable and time interval would be included in the model to adjust for the significant non-proportionate variable. A *p*-value below 0.05 for any variable in the non-proportional excess hazard test indicates a significant non-proportional excess hazard and the variable was considered non-proportionate with the time of diagnosis. The non-proportional excess hazard test is available in the relsurv package. Overdispersion was tested using the deviance statistics against the degrees of freedom of chi-squared distribution and *p*-value below 0.05 indicates a significant overdispersion in the model.

## Results

After exclusion of 49 cases, the remaining 549 cases were included in the analysis. The descriptive statistics were presented in Table 1. For univariable Poisson regression, variable age was subdivided at 50 years old, variable cancer morphology was categorised into two subgroups; infiltrating ductal carcinoma and other types of morphology, and variable ethnicity was categorised into Malay and non-Malay. The result of univariable Poisson regression was presented in Table 2.

**Table 1:** Characteristics of breast cancer cases in Kelantan Cancer Registry (*n* = 549)

Variables	Total n (%)	Died n (%)	Censored n (%)
Survival time (years)^a^	9.9 (0.0, 9.9)	–	–
Survival time (years)^b^	5.4 (6.2)	–	–
Age at diagnosis (years)^c^	50.4 (11.2)	–	–
Age at diagnosis			
> 50 years	248 (45.2)	107 (42.0)	141 (48.0)
≤ 50 years	301 (54.8)	150 (58.0)	151 (52.0)
Ethnicity:			
Malay	471 (85.8)	240 (93.0)	231 (79.0)
Chinese	65 (11.8)	15 (6.0)	50 (17.0)
Indian	5 (1.0)	1 (0.0)	4 (1.0)
Siam	6 (1.1)	0 (0.0)	6 (2.0)
Others	2 (0.4)	1 (0.0)	1 (0.0)
Stages:			
Stage І	163 (29.7)	53 (21.0)	110 (38.0)
Stage ІІ	161 (29.3)	33 (13.0)	128 (44.0)
Stage ІІІ	90 (16.4)	53 (21.0)	37 (13.0)
Stage IV	135 (24.6)	118 (46.0)	17 (6.0)
Morphology:			
Carcinoma, NOS	2 (0.4)	2 (0.8)	0 (0.0)
Carcinoma undifferentiated, NOS	1 (0.2)	0 (0.0)	1 (0.3)
Papillary carcinoma, NOS	17 (3.1)	9 (3.5)	8 (2.7)
Squamous cell carcinoma, NOS	7 (1.3)	4 (1.6)	3 (1.0)
Adenocarcinoma, NOS	68 (12.4)	38 (14.8)	30 (10.3)
Adenoid cystic carcinoma	1 (0.2)	0 (0.0)	1 (0.3)
Infiltrating ductal carcinoma, NOS	448 (81.6)	202 (78.6)	246 (84.3)
Medullary carcinoma, NOS	1 (0.2)	0 (0.0)	1 (0.3)
Lobular carcinoma, NOS	2 (0.4)	1 (0.4)	1 (0.3)
Infiltrating ductal and lobular carcinoma	1 (0.2)	1 (0.4)	0 (0.0)
Sarcoma, NOS	1 (0.2)	0 (0.0)	1 (0.3)
Surgery			
No	275 (50.0)	154 (60.0)	121 (41.0)
Yes	274 (49.9)	103 (40.0)	171 (59.0)
Radiotherapy			
No	444 (80.9)	211 (82.0)	233 (80.0)
Yes	105 (19.1)	46 (18.0)	59 (20.0)
Chemotherapy			
No	386 (70.3)	170 (66.0)	216 (74.0)
Yes	163 (29.7)	87 (34.0)	76 (26.0)

^a^range (minimum value, maximum value)

^b^median (IQR)

^c^mean (SD)

*NOS* not otherwise specified

**Table 2:** Univariable Poisson regression of breast cancer cases in Kelantan Cancer Registry (*n* = 549)

Variables	β coefficient (SE)	Crude EHR (95% CI)	Z statistics	*P*-value
Age at diagnosis^a^				
> 50	0.00	1.00		
≤ 50	0.37 (0.15)	1.44 (1.08, 1.93)	2.47	0.014
Ethnicity^a^				
Non-Malay	0.00	1.00		
Malay	1.28 (0.35)	3.58 (1.81, 7.07)	3.675	< 0.001
Stages^a^				
Stage І	0.00	1.00		
Stage ІІ	−0.65 (0.30)	0.52 (0.29, 0.94)	−2.15	0.031
Stage ІІІ	0.82 (0.23)	2.28 (1.46, 3.54)	3.65	< 0.001
Stage IV	1.89 (0.20)	6.60 (4.50, 9.69)	9.65	< 0.001
Morphology^a^				
Others	0.00	1.00		
Infiltrating ductal carcinoma, NOS	−0.38 (0.17)	0.69 (0.49, 0.96)	−2.20	0.028
Surgery^a^				
No	0.00	1.00		
Yes	−0.84 (−0.15)	0.43 (0.32, 0.58)	−5.61	< 0.001
Radiotherapy^b^				
No	0.00	1.00		
Yes	−0.43 (−0.20)	0.65 (0.44, 0.96)	−2.15	0.031
Chemotherapy^b^				
No	0.00	1.00		
Yes	0.20 (0.15)	1.23 (0.92, 1.64)	1.37	0.170

^a^Time interval used (in year): 0.00, 0.08, 0.17, 0.25, 0.33, 0.42, 0.50, 0.75, 1.00, 1.25, 1.50, 1.75, 2.00, 2.50, 3.00, 3.50, 4.00, 4.50, 5.00, 5.50, 6.00, 7.00, 8.00, 9.00, 10.00

^b^Time interval used (in year): 0.00, 0.08, 0.17, 0.25, 0.33, 0.42, 0.50, 0.75, 1.00, 1.25, 1.50, 1.75, 2.00, 2.50, 3.00, 3.50, 4.00, 4.50, 5.00, 5.50, 6.00, 7.00, 10.00

*EHR* excess hazard ratio, *NOS* not otherwise specified

For multivariable Poisson regression, the final model is presented in Table 3. In this model, variable cancer staging was further categorised into early stage (stage I and II) and late stage (stage III and IV) to ease the convergence of the model. Also, the model included interaction terms between variable surgery and time interval, and variable morphology and time interval, since the excess hazard was not proportionate for both variables. There were no significant two-way interactions between the variables and there was no overdispersion in the model before the adjustment for the significant non-proportional excess hazard (Chi-square (df) = 201.94 (250), *P*-value = 0.989).

**Table 3:** Multivariable Poisson regression of breast cancer cases in Kelantan Cancer Registry (*n* = 549)

Variables	β coefficient (SE)	Adjusted EHR (95% CI)	Z statistic	*P*-value
Age at diagnosis				
> 50	0.00	1.00		
≤ 50	0.39 (0.15)	1.47 (1.11, 1.96)	2.66	0.008
Ethnicity				
Non-Malay	0.00	1.00		
Malay	0.84 (0.29)	2.31 (1.31, 4.09)	2.87	0.004
Stages				
Early stage (stage I and II)	0.00	1.00		
Late stage (stage III and IV)	1.75 (0.16)	5.75 (4.24, 7.81)	11.25	< 0.001
Morphology				
Others	0.00	1.00		
Inf duct CA, NOS	−0.84 (0.21)	0.43 (0.29, 0.64)	−4.09	< 0.001
Surgery				
No	0.00	1.00		
Yes	−1.67 (0.24)	0.19 (0.12, 0.30)	−7.05	< 0.001
Morphology x Time				
Inf duct CA, NOS x Interval 2^b^	1.19 (0.54)	3.30 (1.15, 9.44)	2.23	0.026
Inf duct CA, NOS x Interval 3^c^	1.12 (0.69)	3.06 (0.79, 11.90)	1.62	0.106
Inf duct CA, NOS x Interval 4^d^	0.10 (0.49)	1.11 (0.43, 2.88)	0.21	0.831
Inf duct CA, NOS x Interval 5^e^	0.71 (1.53)	2.02 (0.10, 40.73)	0.46	0.645
Surgery x Time				
Yes x Interval 2^b^	1.23 (0.38)	3.43 (1.63, 7.22)	3.24	0.001
Yes x Interval 3^c^	1.16 (0.48)	3.18 (1.25, 8.07)	2.43	0.015
Yes x Interval 4^d^	2.13 (0.49)	8.44 (3.25, 21.91)	4.39	< 0.001
Yes x Interval 5^e^	0.50 (1.18)	1.64 (0.16, 16.46)	0.42	0.672
Time interval				
Interval 1^a^	−2.02 (0.34)	0.13 (0.07, 0.26)	−5.90	< 0.001
Interval 2^b^	−4.01 (0.58)	0.02 (0.01, 0.06)	−6.95	< 0.001
Interval 3^c^	−4.34 (0.72)	0.01 (0.00, 0.05)	−6.04	< 0.001
Interval 4^d^	−4.68 (0.55)	0.01 (0.00, 0.03)	−8.51	< 0.001
Interval 5^e^	−5.29 (1.44)	0.01 (0.00, 0.08)	−3.68	< 0.001

AIC = 419.38, Deviance = 163.03

Interval 1^a^ = 0–1 years

Interval 2^b^ = 1–2 years

Interval 3^c^ = 2–3 years

Interval 4^d^ = 3–6 years

Interval 5^e^ = 6–10 years

*EHR* excess hazard ratio, *Inf duct CA, NOS* Infiltrating ductal carcinoma, not otherwise specified

Five prognostic factors were found significant in this study were the age at diagnosis, ethnicity, stages, morphology, and surgery. Breast cancer patients diagnosed at age 50 years old and younger had 47% higher excess hazard of death compared to those diagnosed at an older age. Also, Malay breast cancer patients had a 2.31 higher excess hazard of death compared to non-Malay patients. Additionally, late-stage breast cancer patients had a 5.75 higher excess hazard of death than early stage breast cancer patients.

The excess hazard for breast cancer morphology was not proportionate with the time of diagnosis. In Table 3 for example, breast cancer patients with infiltrating ductal carcinoma, not otherwise specified (NOS) in the second interval had a 3.3 higher excess hazard compared to breast cancer patients with infiltrating ductal carcinoma, NOS in the first interval, while those with infiltrating ductal carcinoma, NOS in the fourth interval had only 11% higher excess hazard compared to those with infiltrating ductal carcinoma, NOS in the first interval. The non-proportionate excess hazard effect of breast cancer morphology with the survival time was further categorised in Table 4. The excess hazard for breast cancer patients with infiltrating ductal carcinoma, NOS was lower than those with other types of breast cancer morphology for most of the survival time. However, the excess hazard was higher between one- and three-years following diagnosis. The same occurrence was observed for variable surgery. In Table 3, the breast cancer patients who received surgery in the fourth interval had 8.44 higher excess hazard than breast cancer patients who received surgery in the first interval, while the ratio of the excess hazard of breast cancer patients who received surgery in the fifth interval compared to those in the first interval was only at 64%. The non-proportionate excess hazard effect of the surgery was further categorised in Table 5. Generally, breast cancer patients who received surgery had a lower excess hazard of death than those who did not receive surgery for most of the survival time. However, between period three- and six-years following diagnosis, the patients who received surgery had a higher excess hazard of death than those who did not receive surgery.

**Table 4:** Estimated excess hazard ratio (EHR) for variable morphology separated by time interval for breast cancer cases in Kelantan Cancer Registry

Morphology	Time interval				
	Interval 1 (0–1 year)	Interval 2 (1–2 year)	Interval 3 (2–3 year)	Interval 4 (3–6 year)	Interval 5 (6–10 year)
Others	1.00	1.00	1.00	1.00	1.00
Infiltrating ductal carcinoma, NOS	0.43	1.42	1.32	0.48	0.87

*NOS* Not otherwise specified

**Table 5:** Estimated excess hazard ratio (EHR) for variable surgery separated by time interval for breast cancer cases in Kelantan Cancer Registry

Surgery	Time interval				
	Interval 1 (0–1 year)	Interval 2 (1–2 year)	Interval 3 (2–3 year)	Interval 4 (3–6 year)	Interval 5 (6–10 year)
No	1.00	1.00	1.00	1.00	1.00
Yes	0.19	0.65	0.60	1.59	0.31

## Discussion

This study found that younger breast cancer patients had a higher excess hazard compared to older patients. However, a study in Malaysia found an opposite result in which a higher excess hazard was observed in older breast cancer patients [12], while another study did not find age at diagnosis as a significant prognostic factor of breast cancer [11]. Both studies were population-based studies but used a cause-specific approach in the analysis, which may explain the difference in finding. Additionally, other two hospital-based studies in Malaysia reported that age at diagnosis was not a significant prognostic factor in their study [10, 21]. Both studies used a cause-specific approach in their study design. Our finding, however, is consistent with the other findings that concluded breast cancer patients diagnosed at younger age present with a more advanced and severe tumour thus has a higher risk of mortality [22–25]. Several studies outside Malaysia did find age as a significant prognostic factor of breast cancer. A study done in Singapore using medical records from National Cancer Centre Singapore found that breast cancer patients treated with breast-conserving therapy (BCT) aged 40 years old and below had two times higher risk of mortality compared to those who at an older age [26]. Other two studies in the US found that breast cancer patients who aged 40 years old and below had a higher hazard of death compared those who aged older [27, 28].

Ethnicity was a significant prognostic factor of breast cancer in this current study, which is in agreement with several other studies [10, 11, 21, 29, 30]. Malay breast cancer patients had been observed to present with a more aggressive and larger tumour compared to other ethnic groups [31, 32]. Neighbouring countries such as Singapore also reported a similar finding in which Malay ethnicity is a poor prognostic factor of breast cancer in the country [33]. Additionally, another study done in both Malaysia and Singapore, which used Singapore-Malaysia Breast Cancer Registry found that Malay breast cancer patients had the poorest survival compared to other ethnic groups [32]. A study involving six public hospitals across Malaysia reported that Malay breast cancer patients significantly associated with the use of complementary and alternative medicine (CAM) which had been observed to significantly cause a delay in presentation and diagnosis of breast cancer [34]. Thus, these findings may explain the excess hazard of death observed among Malay breast cancer patients compared to other ethnic groups.

Several studies had reported that cancer staging was a significant prognostic factor of breast cancer [10, 11, 21]. The cancer staging was combined into early stage and late stage in our study due to the convergence issue. A presentation of breast cancer at an advanced stage is a significant contributing factor to breast cancer mortality, especially in low-and-middle-income countries [35]. Also, a similar trend of prognosis of breast cancer in term of cancer staging had been observed in Singapore. According to the Singapore Cancer Registry, between 2011 to 2015, a five-year age-standardised relative survival of breast cancer patients was lowest among patients with stage 4 at 23%, followed by stage 3 at 72%, stage 2 at 89%, and stage 1 at 100% [36]. Additionally, a late-stage presentation of breast cancer in Malaysia may be explained by factors such social and cultural belief, the use of CAM, lack of awareness, and inaccessibility to health care services [37].

Additionally, breast cancer morphology was a significant prognostic factor in this study despite its effect was not proportionate with the time of diagnosis. Other studies had reported that different type of breast cancer such as infiltrating lobular carcinoma (ILC), metaplastic carcinoma of the breast and medullary breast carcinoma had a different survival rate [38, 39]. The non-proportionality of the excess hazard of breast cancer morphology in our study could be explained by factors such as the occurrence of metastases and lymph node involvement. For example, a study done in the United Kingdom had reported that infiltrating ductal carcinoma (IDC) and ILC each had a distinct pattern of lymph node involvement and IDC had a less tendency for metastasis [40]. Unfortunately, this additional information was not available in this study.

Our study found that surgery was a significant prognostic factor of breast cancer, although there was a non-proportionality of excess hazard between surgery and survival time. This finding is consistent with another population-based study in Kelantan despite the difference in the survival analysis approach [11]. Besides, another population-based study done in the East of England had reported a similar finding that surgery is a significant prognostic factor of breast cancer but without the non-proportionality to the survival time [41]. Breast cancer patients who received surgery in the early period following diagnosis most probably those with a more advanced tumour, while in the latter period following diagnosis, those who received a surgery most probably patients who diagnosed with a less advanced tumour. Thus, the difference in the characteristic of breast cancer patients between each time interval may explain the different effect of surgery on breast cancer patients.

Radiotherapy and chemotherapy were not a significant prognostic factor in this study. Several studies had reported a similar finding to ours [8, 11, 42]. On the contrary, a population-based study done in the East of England reported an opposite result to ours in which radiotherapy and chemotherapy were a significant prognostic factor of breast cancer [41]. Additionally, a multicentre study conducted in Malaysia, Singapore, and Hong Kong concluded that adjuvant radiotherapy was associated with survival of breast cancer among patients younger than 40 years old, but not in older patients [43]. Evidently, radiotherapy and chemotherapy had a more complex association involving other types of treatment and factors in which this complexity could not be observed in our population-based data. Admittedly, the majority of breast cancer patients in this study did not receive these two treatments. However, a more focus study in Malaysia should be conducted to evaluate the association between a combination of different type of treatment and breast cancer mortality. So, the benefit of each treatment and in a combination of other treatments could be well observed.

There are a few limitations to our study. Since this study used secondary data from a cancer registry, the information available in this study is, however, limited to the information available in the cancer registry. Important information such as tumour size, degree of metastases, and lymph node involvement was not available. Also, a Poisson regression under the relative survival approach is unable to deal with zero deaths in an interval subgroup. Thus, this leads to difficulty in the model convergence. For example, the levels of cancer staging in our study need to be combined to get a converged model. Lastly, A complete life table of general population mortality was not available for Kelantan population, and therefore, complete life table was expanded from an abridged life table of general population mortality in this study. Other researchers may use a different method of expansion, leading to a lack of standardisation in the relative survival analysis among studies.

## Conclusions

The relative survival approach has been considered as a standard practice among population-based studies, especially in cancer research. This approach provides a better alternative when the cause of death is not reliable or unavailable. A population-based study gives a perspective beneficial for public health planning and policymaking. This population-based study had found three poor prognostic factors significantly associated with breast cancer mortality, which were age below 50 years old, Malay ethnicity, and late stage.

## Data Availability

The General population mortality data for Kelantan that support the findings of this study are available from eStatistik (https://newss.statistics.gov.my/newss-portalx/ep/epLogin.seam). Additionally, the breast cancer data that support the findings of this study are available from Non-communicable Disease (NCD) unit, Kelantan State Health Department but restrictions apply to the availability of these data, which were used under license for the current study, and so are not publicly available. Data are however available from the authors upon reasonable request and with permission of Kelantan State Health Department and Medical Research and Ethics Committee, Ministry of Health Malaysia.

## Abbreviations

AIC: Akaike Information Criterion
BCT: Breast-conserving therapy
CAM: Complementary and alternative medicine
DOSM: Department of Statistics, Malaysia
EHR: Excess hazard ratio
ICD-O: International Classification of Diseases for Oncology
IDC: Infiltrating ductal carcinoma
ILC: Infiltrating lobular carcinoma
IQR: Interquartile range
NCD: Non-Communicable Disease
NOS: Not otherwise specified
Q-Q plot: Quantile-quantile plot
SD: Standard deviation
UKIACR: United Kingdom and Ireland Association of Cancer Registries

## References

[1] Becker S (2015). A historic and scientific review of breast cancer: the next global healthcare challenge. Int J Gynecol Obstet.

[2] Youlden DR, Cramb SM, Yip CH, Baade PD (2014). Incidence and mortality of female breast Cancer in the Asia-Pacific region. Cancer Biol Med.

[3] Azizah AM, Nor Saleha IT, Noor Hashimah A, Asmah ZA, Mastulu W (2015). Malaysian National Cancer Registry Report 2007–2011. Putrajaya.

[4] Mariotto AB, Noone AM, Howlader N, Cho H, Keel GE, Garshell J (2014). Cancer survival: an overview of measures, uses, and interpretation. J Natl Cancer Inst - Monogr.

[5] Dickman PW, Sloggett A, Hills M, Hakulinen T (2004). Regression models for relative survival. Stat Med.

[6] Bhoo-Pathy N, Verkooijen HM, Taib NA, Hartman M, Yip CH (2011). Impact of breast surgery on survival in women presenting with metastatic breast cancer. Br J Surg.

[7] Subramaniam S, Bhoo-Pathy N, Taib NA, Tan GH, See MH, Jamaris S (2015). Breast Cancer outcomes as defined by the estrogen receptor, progesterone receptor, and human growth factor Receptor-2 in a multi-ethnic Asian country. World J Surg.

[8] Balasundram S, Salekan K, Ahmad Shariffuddin FN, Taib NA, Adnan TH (2018). Overall survival and local recurrence among breast Cancer patients in hospital Sultanah Nora Ismail Batu Pahat, 2007-2013. Asian Pacific J Cancer Prev..

[9] Abdullah MM, Mohamed AK, Foo YC, Ling Lee CM, Chua CT, Wu CH (2015). Breast cancer survival at a leading cancer Centre in Malaysia. Asian Pac J Cancer Prev..

[10] Taib NA, Akmal M, Mohamed I, Yip C-H (2011). Improvement in survival of breast cancer patients - trends over two time periods in a single institution in an Asia Pacific country. Malaysia Asian Pac J Cancer Prev.

[11] Nordin N, Yaacob NM, Abdullah NH, Hairon SM (2018). Survival time and prognostic factors for breast Cancer among women in north-east peninsular Malaysia. Asian Pac J Cancer Prev..

[12] Abdullah NA, Wan Mahiyuddin WR, Muhammad NA, Ali ZM, Ibrahim L, Ibrahim Tamim NS (2013). Survival rate of breast cancer patients in Malaysia: a population-based study. Asian Pac J Cancer Prev..

[13] National Cancer Registry Department (2018). Malaysian study on cancer survival (MySCan). Putrajaya.

[14] United Nations (2013). MORTPAK for Windows (Handbook) [POP/SW/MORTPAK/2003].

[15] R Foundation for Statistical Computing. R (2019). A Language and Environment for Statistical Computing.

[16] Ibrahim RI, Ngataman N, Abrisam WNAWM (2017). Forecasting the mortality rates using Lee-Carter model and Heligman-pollard model. J Phys Conf Ser.

[17] Ibrahim RI (2008). Expanding an abridged life table using the Heligman-pollard model. Matematika.

[18] Siran MS, Yusuf MM, Yusoff YS, Basah MYA (2015). Expanding abridge life table by using Heligman pollard method: Malaysian experience 2010-2013. Int J Bus Soc Sci.

[19] Perme MP (2013). relsurv: Relative survival. R package version.

[20] UKIACR (2016). Standard Operating Procedure. Guidelines on Population Based Cancer Survival Analysis This.

[21] Ibrahim NI, Dahlui M, Aina E, Al-Sadat N (2012). Who are the breast Cancer survivors in Malaysia?. Asian Pac J Cancer Prev.

[22] Kheirelseid EAH, Boggs JME, Curran C, Glynn RW, Dooley C, Sweeney KJ (2011). Younger age as a prognostic indicator in breast cancer: a cohort study. BMC Cancer.

[23] McGuire A, Brown JAL, Malone C, McLaughlin R, Kerin MJ (2015). Effects of age on the detection and management of breast cancer. Cancers (Basel).

[24] Tao ZQ, Shi A, Lu C, Song T, Zhang Z, Zhao J (2015). Breast Cancer: epidemiology and etiology. Cell Biochem Biophys.

[25] Assi HA, Khoury KE, Dbouk H, Khalil LE, Mouhieddine TH, El Saghir NS (2013). Epidemiology and prognosis of breast cancer in young women. J Thorac Dis.

[26] Wong FY, Tham WY, Nei WL, Lim C, Miao H (2018). Age exerts a continuous effect in the outcomes of Asian breast cancer patients treated with breast-conserving therapy. Cancer Commun.

[27] Partridge AH, Hughes ME, Warner ET, Ottesen RA, Wong YN, Edge SB (2016). Subtype-dependent relationship between young age at diagnosis and breast cancer survival. J Clin Oncol.

[28] Bharat A, Aft RL, Gao F, Margenthaler JA (2009). Patient and tumor characteristics associated with increased mortality in young women (≤40 years) with breast cancer. J Surg Oncol.

[29] Sung H, Devi CRB, Guida J, Tang TS, Anderson WF, Yang XR (2016). Abstract 3414: Ethnic disparities in breast cancer survival in Sarawak, Malaysia. AACR 107th Annual Meeting 2016.

[30] Al-Naggar RAM, Md Isa Z, Shah SA, Mohd Nor MI, Chen R, Ismail F (2009). Eight year survival among breast cancer Malaysian women from university Kebangsaan Malaysia medical Centre. Asian Pacific J Cancer Prev.

[31] Yip CH, Teo S, Bhoo-Pathy N (2014). A review of breast cancer research in Malaysia. Med J Malaysia.

[32] Bhoo-Pathy N, Hartman M, Yip C-H, Saxena N, Taib NA, Lim S-E (2012). Ethnic differences in survival after breast Cancer in South East Asia. PLoS One.

[33] Xin WR, Kwok L, Yong WF (2017). Screening uptake differences are not implicated in poorer breast Cancer outcomes among Singaporean Malay women. J Breast Cancer.

[34] Mujar NMM, Dahlui M, Emran NA, Hadi IA, Wai YY, Arulanantham S (2017). Complementary and alternative medicine (CAM) use and delays in presentation and diagnosis of breast cancer patients in public hospitals in Malaysia. PLoS One.

[35] Unger-Saldaña K (2014). Challenges to the early diagnosis and treatment of breast cancer in developing countries. World J Clin Oncol.

[36] National Registry of Diseases Office (2017). Singapore Cancer Registry Annual Registry Report 2015.

[37] Cheng ML, Ling DY, Nanu PKP, Nording H, Lim CH (2015). Factors influencing late stage of breast cancer at presentation in a district hospital - Segamat hospital. Johor Med J Malaysia.

[38] Lai HW, Tseng LM, Chang TW, Kuo YL, Hsieh CM, Chen ST (2013). The prognostic significance of metaplastic carcinoma of the breast (MCB) - a case controlled comparison study with infiltrating ductal carcinoma. Breast.

[39] Cao A-Y, He M, Huang L, Shao Z-M, Di G-H (2013). Clinicopathologic characteristics at diagnosis and the survival of patients with medullary breast carcinoma in China: a comparison with infiltrating ductal carcinoma-not otherwise specified. World J Surg Oncol.

[40] Fernández B, Paish EC, Green AR, Lee AHS, Macmillan RD, Ellis IO (2011). Lymph-node metastases in invasive lobular carcinoma are different from those in ductal carcinoma of the breast. J Clin Pathol.

[41] Ali AMG, Greenberg D, Wishart GC, Pharoah P (2011). Patient and tumour characteristics, management, and age-specific survival in women with breast cancer in the east of England. Br J Cancer.

[42] Cihan YB, Arslan A, Cetindag MF, Mutlu H (2014). Lack of prognostic value of blood parameters in patients receiving adjuvant radiotherapy for breast Cancer. Asian Pac J Cancer Prev..

[43] Bhoo-Pathy N, Verkooijen HM, Wong FY, Pignol JP, Kwong A, Tan EY (2015). Prognostic role of adjuvant radiotherapy in triple-negative breast cancer: a historical cohort study. Int J Cancer.

[44] Hanis TM, Yaacob NM, Hairon SM, Abdullah S, Nordin N, Abdullah NH (2019). Modelling Excess Mortality Among Breast Cancer Patients in the North East Region of Peninsular Malaysia, 2007–2011: A Population-Based Study.

